# A Multi-center Simulation-Based Emergency Medicine Resident Boot Camp Can Improve Teamwork, Communication, and Clinical Skills

**DOI:** 10.7759/cureus.55083

**Published:** 2024-02-27

**Authors:** Richard Shin, Kent Li, Kevin Hon, Kevin Ching, Timothy C Clapper

**Affiliations:** 1 Emergency Medicine, New York-Presbyterian Queens, New York, USA; 2 Pediatric Emergency Medicine, Weill Cornell Medical College, New York, USA; 3 Pediatrics, Weill Cornell Medicine NewYork-Presbyterian Simulation Center, New York, USA; 4 Pediatrics, Weill Cornell Medical College, New York, USA

**Keywords:** skills and simulation training, acgme core competencies, emergency medicine resident, boot camps, • simulation in medical education

## Abstract

Boot camps are designed to deliver highly specific education in a short amount of time. Educational boot camps are known to improve confidence in clinical capabilities and medical knowledge and promote teamwork skills. We created an emergency medicine (EM) boot camp with targeted learning objectives based on expected mastery of post-graduate year (PGY)-level educational objectives based on the Accreditation Council for Graduate Medical Education (ACGME) EM milestones. This boot camp included a qualitative assessment, survey-based feedback, and quantitative assessment, which included the team's performance utilizing a validated code team checklist (Cardiac Code Management Assessment Tool). After attending the conference, EM residents felt more confident in achieving the EM ACGME milestones including the ability to provide immediate interventions to a critical patient, effective use of team communication, the ability to switch tasks efficiently, and to provide real-time feedback to their team. Eighty-six percent of residents preferred this teaching modality over other conference-based didactics and would like to see greater incorporation of similar interventions in future conferences.

## Introduction

Medical simulation is an instrumental teaching modality for learners throughout the medical field [1-5]. One of the more recent developments in medical simulation is the implementation of educational boot camps. This model efficiently delivers specific educational content in a short period of time. Many boot camps have been shown to have a qualitative impact on confidence in clinical capabilities, medical knowledge, and promoting teamwork skills [6-10]. There is, however, less robust research on the effectiveness of simulation boot camps on quantitative metrics. Moreover, there is a notable heterogeneity in the approach to resuscitation in the most critically ill patients. To provide a framework for learners when caring for unstable patients, we have devised a tiered, simulation-based resuscitation boot camp for Emergency Medicine (EM) residents. This study aims to establish resuscitation fundamentals for learners that are specific to different post-graduate year (PGY) levels, and in the process, promote an improvement of skills in teamwork and communication.

There are many advantages to the boot camp method of education delivery. By definition, boot camps are low-stakes formative educational events. They have the potential to be highly efficient, being administered over several short and focused sessions. They can be immersive, especially when combined with the use of hands-on task trainers and simulated patient scenarios. They are also collaborative, as a platform for learners and teachers of varied experiences to come together. However, they may be too broad of a scope and difficult to individualize for each learner’s level. In an attempt to remedy this issue, we employed a simulation-based resuscitation boot camp with educational small-group sessions specific to the PGY level. We hoped that by employing tiered learning objectives we could allow for a more targeted boot camp experience and promote improvement that is commensurate to a learner’s level of expected mastery.

The Accreditation Council for Graduate Medical Education (ACGME) milestones are competency-based developmental outcomes that can be demonstrated progressively by residents from the beginning of their education through graduation to the unsupervised practice of their specialties. Similar to prior studies, we utilized a qualitative survey with questions derived from relevant ACGME milestones to evaluate the impact of this intervention. A boot camp experience has proven to be qualitatively effective as an educational modality and demonstrates a correlation with satisfaction for medical trainees [11,12]. We hoped to better identify areas of improvement for longitudinal resident developmental education through self-evaluation of resident learners’ perceived abilities [13]. In another effort to assess the possible impact on quantitative measures during resuscitation, we also employed a validated code team checklist as a part of the intervention to assess the degree of behavioral changes after the intervention.

Importantly, as an educational tool, boot camps offer the ability to establish a safe learning environment. This encourages collaboration among residents and educators, establishes a fun learning environment, and ultimately may promote progress toward the achievement of milestones. By leveraging tiered PGY-specific learning objectives during the boot camp, we hypothesize that residents will make improvements in self-perceived ACGME milestones within resuscitation. Furthermore, we believe the intervention will allow residents to apply their learning toward improved scores on a quantitative code team checklist.

Here, we present a multi-center EM boot camp, conducted in New York City, focused on PGY-specific learning objectives comprised of educational sessions followed by a concluding medical simulation team-based competition with participants from each campus. An observational assessment using mixed methods of both quantitative and qualitative metrics was used to determine the efficacy of the boot camp concerning the validated Cardiac Code Management Assessment Tool (Appendix B; Table 8 and Table 9) and self-assessment of EM ACGME Milestones. Qualitative metrics were obtained by survey to assess the impact on resident wellness and learning preference.

## Materials and methods

As a part of this study, we collaborated with two other EM residencies in our network, to create a boot camp educational delivery method. It differs from prior interventions in specifying targeted learning objectives based on expected mastery according to PGY level. Moreover, simulation is utilized throughout the boot camp to allow for the application of learning. This study also assessed quantitative performance using a validated code team checklist. The development of this formative assessment was constructed with key medical simulation and EM faculty at three academic EM campuses. A panel of six EM faculty members with a background in medical education identified relevant ACGME milestones (Table 1) on resuscitation and created a qualitative self-assessment survey (Appendix C, Table 10). Our group then developed small group topics for each PGY class based on the identified milestones (Table 2).

**Table 1:** ACGME EM milestones surveyed in the study. A self-assessment survey was extrapolated based on the identified milestones. EM, emergency medicine; ACGME, Accreditation Council for Graduate Medical Education; PC, patient care; IC, interpersonal communication; SBP, systems-based practice

Self-assessment of relevant ACGME milestones	
Patient care 1 (PC-1)	Emergency stabilization
Patient care 7 (PC-7)	Task switching
Patient care 8 (PC-8)	Approach to procedures - indications and benefits
Interpersonal communication 1 (IC-1)	Patient and family communication
Interpersonal communication 2 (IC-2)	Interprofessional and team communication
Systems-based practice 3 (SBP-3)	System navigation for patient-centered care

**Table 2:** Listed are the topics for each small group. Residents were divided according to PGY level to allow for more targeted learning objectives. PGY, post-graduate year; CPR, cardiopulmonary resuscitation; ECMO, extracorporeal membrane oxygenation; TEE, transesophageal echocardiogram

PGY-specific breakout groups		
PGY-I	Airway essentials	
	High-quality CPR, shockable rhythms and defibrillation	
	Bradycardia algorithm, transcutaneous pacing	
PGY-II	Difficult airway algorithm and cricothyrotomy	
	Identifying reversible causes of death and pericardiocentesis	
	Communication and leadership during resuscitation	
PGY-III/IV	ECMO indications and considerations	
	Goals of care discussions	
	Fiberoptic intubation	
	TEE essentials	

Residents of PGY levels 1 through 4 from these three EM residencies participated in a one-day, simulation-based boot camp intervention (Appendix A, Tables 4-7). Residents recruited as study participants on the day of the educational event and those who did not wish to voluntarily participate in the educational study were excluded.

**Figure 1:**
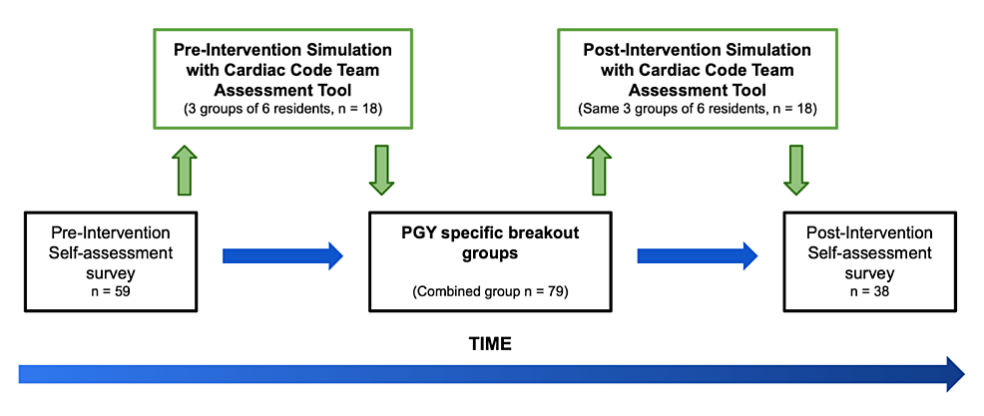
A subset of the cohort was assessed in their performance during simulated cases using the validated Cardiac Code Team Assessment Tool both before and after the small group sessions. PGY, post-graduate year

At the start of the day, residents filled out a qualitative self-assessment survey based on a five-point Likert scale, in order to assess their perceived proficiency in relevant ACGME milestones. Next, the EM residents were divided based on PGY level for educational small group sessions specific to their expected level of knowledge. Each small group included an introductory medical simulation, followed by hands-on practice and didactics. The sessions were administered over approximately 30 minutes and led by an EM faculty instructor who debriefed the learners and proctored didactic discussions. After completing the compendium of educational small groups, residents again completed a post-intervention qualitative self-assessment survey (Figure 1) (Appendix D, Table 11). Included as part of the final survey were questions on resident attitudes and preferences on the delivery of educational curricula.

For the quantitative portion, three groups of six EM residents, one from each participating campus (n=18), were evaluated for performance during a simulated cardiac arrest using the validated Cardiac Code Management Assessment Tool (Appendix B) checklist, in both pre- and post-intervention. Two independent faculty observers scored each group using the checklist. Simulation cases were standardized to case stem, events, proctor, and specific time points among all groups.

## Results

**Figure 2:**
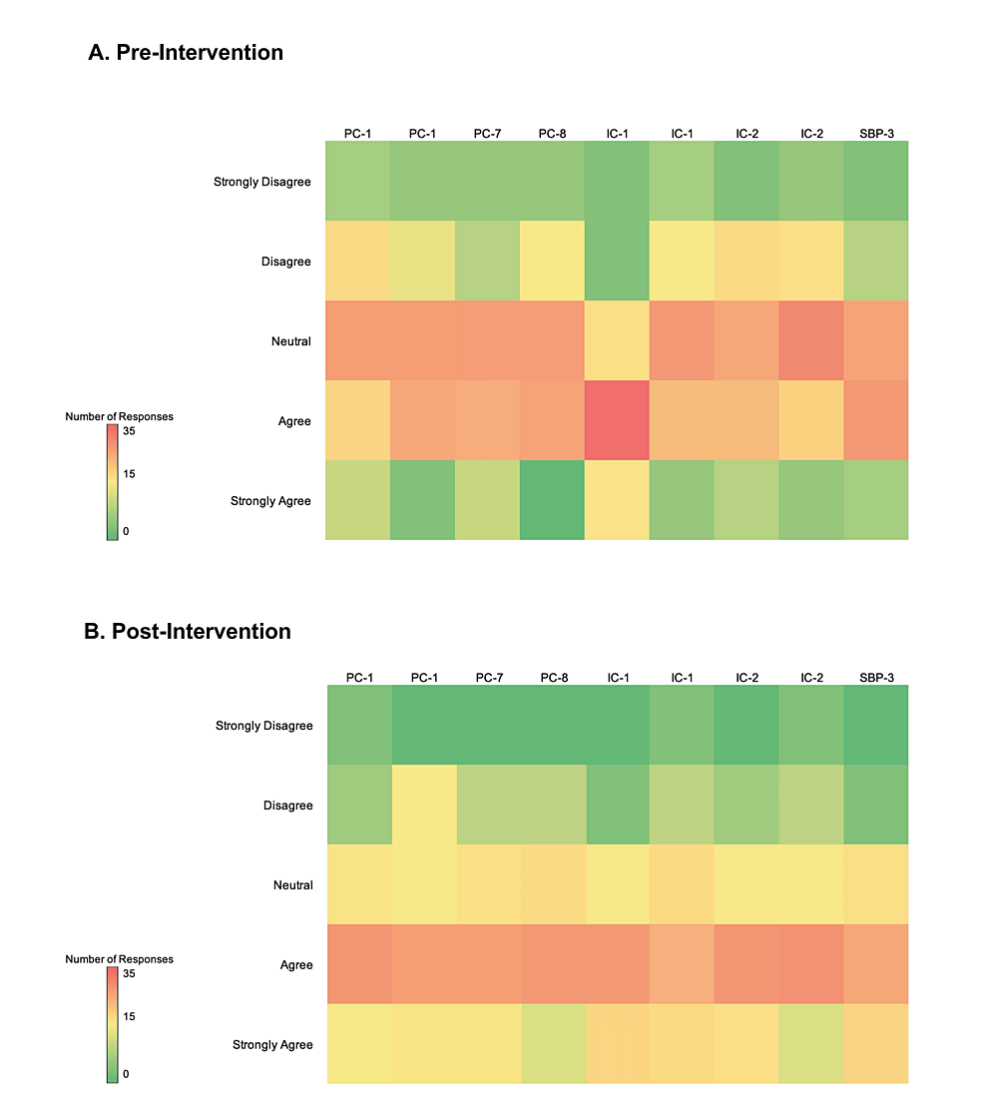
Heatmaps display pre-intervention and post-intervention qualitative survey data (A and B) for relevant ACGME milestones assessed based on a five-point Likert scale. PC, patient care; IC, interpersonal communication; SBP, systems-based practice

A total of 79 total learners participated in the study. The breakdown of our study sample included 21 learners at PGY-3/4, 18 at PGY-2, and 18 at PGY-1 level. During the qualitative portion of the study, the total number of respondents to the survey was 59 pre-intervention and 38 post-intervention (Appendix C, Table 10; Appendix D, Table 11). Pre-intervention scores reflected that residents scored a mode of three, corresponding with neutral confidence for most milestones. This was improved to a mode of four following the curriculum, corresponding to improved self-confidence in a majority of milestones. There appeared to be the largest improvement of confidence in the ability to apply immediate interventions to a critical patient, effective use of team communication, ability to switch tasks efficiently, and to provide real-time feedback. Overall, most residents felt more confident in their ability to achieve relevant milestones following the resuscitation boot camp (Figure 2).

The quantitative arm of the study was analyzed using a paired sample t-test, the three groups scored a mean of 24.7 (SD = 3.5) during the pre-intervention cardiac arrest simulation. During the post-intervention simulation, the score averaged 28.7 (SD = 2.3), with a mean improvement of 3.0 points (P = 0.25) (Table 3). An analysis of the data using two-way ANOVA showed no significant difference in the overall score using the Cardiac Code Management Tool checklist between sites (P = 0.54), as well as pre- and post-intervention (P = 0.25).

**Table 3:** Listed are the absolute scores based on the Cardiac Code Management Assessment Tool checklist for each of the participating sites.

Cardiac Code Management Assessment Tool scores		
	Pre-intervention	Post-intervention
Team A	21	30
Team B	26	30
Team C	25	26

A portion of the post-intervention survey also polled EM residents on attitudes toward a boot camp approach to learning. All learners enjoyed participating in the educational intervention. Thirty-three respondents (86%) also preferred this teaching modality over traditional lecture-based didactics and would like to see greater incorporation of similar interventions in future conferences. Thirty-five respondents either agreed or strongly agreed that the simulation-based education was enjoyable and offered a benefit to professional well-being.

## Discussion

Our boot camp intervention provided evidence that a targeted approach can offer improved self-perception of proficiency in resuscitation. The qualitative data is limited to selected milestones and, therefore, should be interpreted with caution. It is notable that the selected milestones were identified as immediately relevant to resuscitation by experienced faculty members and may not capture the entirety of the skills required. Moreover, another limitation is in the point that self-perception of relevant ACGME milestones has not been verified to correlate with actual performance. The milestones were devised to be achieved over the course of an entire residency training period and thus may be reductive to extrapolate proficiency to a one-day boot camp. Despite these limitations, this qualitative survey represents an exceptionally meaningful concept to guide and model teaching objectives.

One of the limitations of the study is the low sample size when performing the cardiac code assessment. The ability to record additional iterations of quantitative checklist data was limited by time constraints during the one-day intervention. There were only three groups assessed before and after the curriculum. Due to this limited sample, improvements in absolute scores during the cardiac code assessment checklist did not reach statistical significance. Additionally, selection bias may have influenced the quantitative dataset. Of the selected groups, individual party members were noted to have variable PGY training years. Based on their pre-existing experience, there may be a different baseline level of medical knowledge and exposure to prior resuscitation of cardiac arrest. Future studies in this area should seek to assess participants in these areas before participation.

Another area of improvement during the checklist data collection would be to expand the number of independent faculty graders. Due to the availability of qualified faculty, only two observers were available for data collection. Additional faculty observers would help enhance the robustness of collected data and improve inter-rater reliability.

During the post-intervention qualitative survey, it was noted that the dropout rate was higher than anticipated. A total of 21 respondents filed a pre-intervention survey but did not complete the post-intervention survey. This was largely due to numerous residents exiting the session prematurely due to scheduled clinical obligations. An attempt for improved compliance with this data was built into our intervention through the use of QR codes, as well as email reminders.

## Conclusions

Overall, a simulation-based resuscitation boot camp with tiered, PGY-specific learning objectives is a feasible and effective teaching modality. Through this study, there is evidence that a targeted approach can promote advances in skill levels that is better tailored to a learner’s level of mastery. Further studies are needed to evaluate if boot camps can improve a participant's self-perceived proficiency in EM ACGME milestones. While there does not appear to be a statistically significant difference in checklist score after the one-day EM resident-based boot camp, there is an improvement in the absolute score on the Cardiac Code Team Assessment Tool for each assessed group. Moreover, additional qualitative survey feedback suggests that this simulation-based boot camp is largely preferred over traditional teaching conference models and can contribute to a safe learning environment in our study cohort.

Our targeted approach has shown promise in helping to standardize resuscitation goals for specific levels of learner experience. One of the most compelling aspects of this model is the ability to create a safe learning environment during small group sessions by grouping learners with a similar knowledge base. Additionally, we were able to create an environment where interfacility faculty engagement and collaboration were supported. This approach furthermore allows for a platform for learners to synthesize these individual learning objectives and to apply their collective skills through simulation. Future directions could include similar annual tiered boot camps covering topics including point of care ultrasound, obstetrics and pediatric emergencies, and urgent care procedures. We hope that this tiered approach can be a teaching model forward, not just to EM residents, but to other medical team members and specialties.

## Appendices

Appendix A

**Table 4:** Schedule for emergency medicine resident boot camp. PGY, post-graduate year

Timeline	
7:30 AM - 8:00 AM	Qualitative data collection - three small teams
8:00 AM - 8:20:00 AM	Introduction and survey distribution to the larger group
8:30 AM - 11:00 AM	Educational intervention by PGY level year
11:00 AM - 11:15AM	Qualitative survey-based data collection for the larger group
11:15 AM - 12:45 PM	Educational intervention with quantitative data collection on the same 3 teams as above
12:45 PM - 1:00PM	Final qualitative survey for the larger group

**Table 5:** Schedule for PGY-1 EM resident boot camp. EM, emergency medicine; PGY, post-graduate year; ETT, endotracheal tube; BVM, bag valve mask; OPA, oral pharyngeal airway, NPA, nasopharyngeal airway, ETCO_2_, end-tidal carbon dioxide; CPR, cardiopulmonary resuscitation; TCP, transcutaneous pacemaker; BRASH, bradycardia renal failure, AV blocker, shock, hyperkalemia; ECMO, extracorporeal membrane oxygenation

	Room	Learning objective	Learners	# of learners
8:00 AM - 8:20:00 AM	Auditorium	Introduction: why?, purpose, who, learning objectives, format for the day, split into mixed groups	All	
8:30 AM - 9:05 AM; 1st	B corridor F	ETT and endotracheal intubation	PGY-1-A1+A2	7
8:30 AM - 9:05 AM; 1st	B corridor A	Ventilation: BVM, OPA, NPA, ETCO2, prep for intubation	PGY 1-B1+B2	7
8:30 AM- 9:05 AM; 1st	B corridor B	High-quality CPR, defibrillation, increase chest compression fraction	PGY 1-C1	4
8:30 AM - 9:05 AM; 1st	B corridor C	High-quality CPR, defibrillation, increase chest compression fraction	PGY 1-C2	3
8:30 AM - 9:05 AM; 1st	B corridor D	TCP, bradycardia algorithm, BRASH, hypoxia	PGY 1-D1	3
8:30 AM - 9:05 AM; 1st	B corridor E	TCP, bradycardia algorithm, BRASH, hypoxia	PGY 1-D2	3
9:10 AM - 9:45 AM; 2nd	B corridor F	ETT and endotracheal intubation	PGY-1-B1+B2	10
9:10 AM - 9:45 AM; 2nd	B corridor A	Ventilation: BVM, OPA, NPA, ETCO2, prep for intubation	PGY 1-C1+C2	10
9:10 AM - 9:45 AM; 2nd	B corridor B	High-quality CPR, defibrillation, increase chest compression fraction	PGY 1-D1	5
9:10 AM - 9:45 AM; 2nd	B corridor C	High-quality CPR, defibrillation, increase chest compression fraction	PGY 1-D2	5
9:10 AM - 9:45 AM; 2nd	B corridor D	TCP, bradycardia algorithm, BRASH, hypoxia	PGY 1-A1	5
9:10 AM - 9:45 AM; 2nd	B corridor E	TCP, bradycardia algorithm, BRASH, hypoxia	PGY 1-A2	5
9:50 AM - 10:25 AM; 3rd	B corridor F	ETT and endotracheal intubation	PGY-1-C1+C2	10
9:50 AM - 10:25 AM; 3rd	B corridor A	Ventilation - BVM, OPA, NPA, ETCO2, prep for intubation	PGY 1-D1+D2	10
9:50 AM - 10:25 AM; 3rd	B corridor B	High-quality CPR, defibrillation, increase chest compression fraction	PGY 1-A1	5
9:50 AM - 10:25 AM; 3rd	B corridor C	High-quality CPR, defibrillation, increase chest compression fraction	PGY 1-A2	5
9:50 AM - 10:25 AM; 3rd	B corridor D	TCP, bradycardia algorithm, BRASH, hypoxia	PGY 1-B1	5
9:50 AM - 10:25 AM; 3rd	B corridor E	TCP, bradycardia algorithm, BRASH, hypoxia	PGY 1-B2	5
10:30 AM - 11:05 AM	B corridor F	ETT and endotracheal intubation	PGY-1-D1+D2	10
10:30 AM - 11:05 AM; 4th	B corridor A	Ventilation - BVM, OPA, NPA, ETCO2, prep for intubation	PGY 1-A1+A2	10
10:30 AM - 11:05 AM; 4th	B corridor B	High-quality CPR, defibrillation, increase chest compression fraction	PGY 1-B1	5
10:30 AM - 11:05 AM; 4th	B corridor C	High-quality CPR, defibrillation, increase chest compression fraction	PGY 1-B2	5
10:30 AM - 11:05 AM; 4th	B corridor D	TCP, bradycardia algorithm, BRASH, hypoxia	PGY 1-C1	5
10:30 AM - 11:05 AM; 4th	B corridor E	TCP, bradycardia algorithm, BRASH, hypoxia	PGY 1-C2	5
11:05 AM - 11:15 AM	Break	Auditorium		
11:15 AM - 12:45 PM	Auditorium	Simwars- 3 separate cases	All	
		Case 1: Drowning - ECMO, CPR, intubation		
		Case 2: Grill explosion - difficult airway algorithm, CPR		
		Case 3: Type A dissection, elderly (ECMO) pericardiocentesis, goals of care discussion		
12:45 PM - 1:00 PM	Voting/conclusion			

**Table 6:** Schedule for PGY-2 EM resident boot camp. EM, emergency medicine; CASA, cardiac arrest sonographic assessment; CPR, cardiopulmonary resuscitation; PGY, post-graduate year; ECMO, extracorporeal membrane oxygenation

	Room	Learning objective	Learners	# of learners
8:00 AM - 8:20:00 AM	Auditorium	Introduction - Why?, purpose, who, learning objectives, format for the day, split into mixed groups	All	
8:30AM - 9:15 AM	B corridor D	Blindfolded leader - communication/leadership	PGY-2-A1+A2	10
8:30AM - 9:15 AM	B corridor E	Difficult airway algorithm - cricothyrotomy	PGY-2-B1	5
8:30AM - 9:15 AM	B corridor G	Difficult airway algorithm - cricothyrotomy	PGY-2-B2	5
8:30AM - 9:15 AM	B corridor H	Identify reversible causes with CASA - pericardiocentesis	PGY-2-C1	5
9:20AM - 10:05 AM	B corridor D	Blindfolded leader - communication/leadership	PGY-2-B1+B2	10
9:20AM - 10:05 AM	B corridor E	Difficult airway algorithm – cricothyrotomy	PGY-2-C1	5
9:20AM - 10:05 AM	B corridor G	Difficult airway algorithm – cricothyrotomy	PGY-2-C2	5
9:20AM - 10:05 AM	B corridor H	Identify reversible causes with CASA - pericardiocentesis	PGY-2-A1	5
10:10 AM - 10:55 AM	B corridor D	Blindfolded leader - Communication/leadership - selective attention video	PGY-2-C1+C2	10
10:10 AM - 10:55 AM	B corridor E	Difficult airway algorithm - cricothyrotomy	PGY-2-A1	5
10:10 AM - 10:55 AM	B corridor G	Difficult airway algorithm - cricothyrotomy	PGY-2-A2	5
10:10 AM - 10:55 AM	B corridor H	Identify reversible causes with CASA - pericardiocentesis	PGY-2-B1	5
11:00AM - 11:15AM	Break		Auditorium	
11:15AM - 12:45 PM	Auditorium	Simwars- 3 separate cases	All	
		Case 1: Drowning - ECMO, CPR, intubation		
		Case 2: Grill explosion - difficult airway algorithm, CPR		
		Case 3: Type A dissection, elderly (ECMO) pericardiocentesis, when to call the code		
12:45PM - 1:00PM	Voting/conclusion			

**Table 7:** Schedule for PGY-2 EM resident boot camp. CPR, cardiopulmonary resuscitation; PGY, post-graduate Year; ECMO, extracorporeal membrane oxygenation

8:00 AM - 8:20:00 AM	Auditorium	Introduction - why?, purpose, who, learning objectives, format for the day, split into mixed groups	All	
8:30 AM - 9:05 AM; 1st	B corridor F	ECMO Sim - when we should consider ECMO in cardiac arrest	PGY 3-A	5
	B corridor G	ECMO Sim - when we should consider ECMO in cardiac arrest	PGY 3-A	5
	B corridor H	Respiratory failure opiate and DNR/DNI	PGY 3-B	5
	F corridor F1	Respiratory failure opiate and DNR/DNI	PGY 3-B	5
	F corridor F2	When to call a code	PGY 3-C	5
	F corridor F4	When to call a code	PGY 3-C	5
	F corridor F5	Intro to transesophageal echocardiogram	PGY 3-D	10
9:10 AM - 9:45 AM; 2nd	B corridor F	ECMO Sim - when we should consider ECMO in cardiac arrest	PGY 3-B	5
	B corridor G	ECMO Sim - when we should consider ECMO in cardiac arrest	PGY 3-B	5
	B corridor H	Respiratory failure opiate and DNR/DNI	PGY 3-C	5
	F corridor F1	Respiratory failure opiate and DNR/DNI	PGY 3-C	5
	F corridor F2	When to call a code	PGY 3-D	5
	F corridor F4	When to call a code	PGY 3-D	5
	F corridor F5	Intro to transesophageal echocardiogram	PGY 3-A	10
9:50 AM - 10:25 AM; 3rd	B corridor F	ECMO Sim - when we should consider ECMO in cardiac arrest	PGY 3-C	5
	B corridor G	ECMO Sim - when we should consider ECMO in cardiac arrest	PGY 3-C	5
	B corridor H	Respiratory failure opiate and DNR/DNI	PGY 3-D	5
	F corridor F1	Respiratory failure opiate and DNR/DNI	PGY 3-D	5
	F corridor F2	When to call a code?	PGY 3-A	5
	F corridor F4	When to call a code?	PGY 3-A	5
	F corridor F5	Intro to transesophageal echocardiogram	PGY 3-B	10
10:30 AM - 11:05 AM; 4th	B corridor F	ECMO Sim - when we should consider ECMO in cardiac arrest	PGY 3-D	5
	B corridor G	ECMO Sim - when we should consider ECMO in cardiac arrest	PGY 3-D	5
	B corridor H	Respiratory failure opiate and DNR/DNI	PGY 3-A	5
	F corridor F1	Respiratory failure opiate and DNR/DNI	PGY 3-A	5
	F corridor F2	When to call a code?	PGY 3-B	5
	F corridor F4	When to call a code?	PGY 3-B	5
	F corridor F5	Intro to transesophageal echocardiogram	PGY 3-C	10
11:05 AM - 11:15 AM	Break	Auditorium		
11:15 AM - 12:45 PM	Auditorium	Simwars - 3 separate cases (15 Min w 15 Min Debreifing): summer time	All	
		Case 1: Drowning		
		Case 2: Grill explosion		
		Case 3: Non-exertional heat stroke		
12:45 PM - 1:00 PM	Voting/conclusion			

 Appendix B

**Table 8:** Cardiac Code Management Assessment Tool (1 of 2). CPR- CardioPulmonary Resuscitation, BVM- Bag Valve Mask, PVT- Pulseless Ventricular Tachycardia, VF- Ventricular Fibrillation, IO-Intraosseous, SBAR-Situation, Background, Assessment, Recommendation, IV- Intravenous

Part I – Code Team Skills (Task order may not imply proper sequence) Time from call to arrival: _________________________________________ Shift: Weekday / Weekend (circle) Time from call to first compressions: _______________________________ Day / Overnight (circle)			
Task	Criteria	Not done = 0 points; partly done = 1; point done well = 2 points	
Assess patient/recognition	States the problem clearly and calmly (e.g, “We do not have a pulse.”)		
Start CPR	Compressions started in less than one minute of recognizing a pulseless patient		Time:__________
High-quality CPR performed consistently (every team member)	Follows C-A-B; Correct depth (2 - 2 1/2 inches for adults) and rate (no more or less than 100-120 w/ full recoil); pauses minimized. Add 1 point if CPR board is placed under the patient within three CPR cycles		
CPR improvement	Lowers bed and/or uses step stool to ensure/improve CPR quality		
Airway cleared/established	Considers nasal and oral airways to increase the success of BVM (if verbalizes need or there is airway resistance and does not use: zero points)		
Attach monitor/defibrillator	Defibrillator pads are attached if not already performed by a nurse. Defibrillation is delivered in less than 3 minutes of identifying a shockable rhythm (PVT/VF). Uses correct size and placement		Time:__________
Checks IV/IO access	If peripheral IV access is not achieved within 90 secs or two attempts, the IO access is initiated Add 1 pt for verbalizing request for IO device to initiate access, if needed.		
Code activities are recorded	Team activities and medications are annotated on the code flow sheet within one minute after 5-6 people have arrived		
2-person CPR	2 person CPR with 30:2 compressions started as soon as shaded items are performed or team composition allows		
Team leader follows ACLS algorithms	Determines if advanced airway and additional assistance are needed (i.e., respiratory therapy, radiology tech, etc.)		

**Table 9:** Cardiac Code Management Assessment Tool (2 of 2). IV, intravenous

Part II - teamwork and communication		
	Not Done = 0 points; partly done = 1 point; done well = 2 points	Notes/examples
There is a consistent, clear leader (announced or very clear)		
SBAR provided frequently to team members entering the case		
Leader directly assigns roles or team members self-assign and announce		
Time keeper/recorder directly assigned or self-assigned and announced		
Leader engaged and then positioned where they can control team and see patient information		
Leader summarizes and shares thoughts out loud to establish/reestablish shared mental model and elicit input from team members		Time:__________
Team members demonstrate effective closed-loop communication (request(s) followed through to completion and leader/team informed with a call out) (e.g., “Let’s get an IV in him,” “Starting IV,” “IV is in”)		
Team members apply check-backs as needed (“Give the first dose of 1 cc of Epi” Check-back: “Do you mean 1 mg?”)		
Team members apply mutual support in the form of callouts and task assistance based on environmental scan (STEP: Status of the patient, team members, environment, progress toward the goal)		
If environment is noisy and team members talk over each other/conversations are disconnected, deduct 2 pts.		
Leader conducts team debriefing (If queued, state only, “is there anything you want to say to your team?”) - order or events -what went well and can be improved - identification of resources needed for similar cases in the future or systems issues that need to be reported (near misses, safety concerns)		
		Part 1 Code Team Skills___________ Part 2 Teamwork and Communication__________ TOTAL SCORE__________

Appendix C 

**Table 10:** Multi-center EM resident boot camp pre-survey questions. EM, emergency medicine

Multi-center EM resident boot camp pre-survey questions
What is your level of training:
I feel confident in applying immediate interventions to manage a critically ill patient.
I am confident in my ability to task-switch efficiently during a medical resuscitation.
I am confident in my ability to use effective communication to manage a team during medical resuscitation.
I am confident in my ability to provide real-time feedback to my team members during a medical resuscitation.
I am confident in using shared decision-making with a patient to align their preferences with potential treatment options.
During high-risk situations, I am confident in my ability to recognize indications, risks, benefits, and alternatives for each procedure.
When caring for critically ill patients, I am confident in my ability to provide effective transitions of care/hand-offs.
I am confident in my ability to recognize when an intervention is futile for a patient.
I am confident in my ability to discuss advanced care directives with patients/ family during an emergent situation.

 Appendix D

**Table 11:** Multi-center emergency medicine resident boot camp post-survey questions.

Multi-center emergency medicine resident boot camp post-survey questions
What is your level of training
I feel confident in applying immediate interventions to manage a critically ill patient
I am confident in my ability to task-switch efficiently during a medical resuscitation
I am confident in my ability to use effective communication to manage a team during a medical resuscitation
I am confident in my ability to provide real-time feedback to my team members during a medical resuscitation
I am confident employing flexible communication strategies to resolve conflict among healthcare team members
I am confident in using shared decision making with a patient to align their preferences with potential treatment options
During high-risk situations, I am confident in my ability to recognize indications, risks, benefits, and alternatives for each procedure
When caring for critically ill patients, I am confident in my ability to provide effective transitions of care/hand-offs
I am confident in my ability to recognize when an intervention is futile for a patient
I am confident in my ability to discuss advanced care directives with patients/ family during an emergent situation
I enjoyed participating in simulation-based education and in SimWars
I am comfortable learning in simulation-based education and in SimWars
Simulation based education and SimWars is preferred over my usual conference style
I would like to integrate more conference days like today
I feel more confident in my abilities to lead a resuscitation
Compared to traditional conference, simulation-based education and SimWars offered a greater benefit to my professional well-being and overall wellness
I feel burnt out due to my work
Getting together with others outside of my residency in simulation-based education and in SimWars improves my overall wellness
Additional feedback or comments
